# Polyproline and Triple Helix Motifs in Host-Pathogen Recognition

**DOI:** 10.2174/138920312804871157

**Published:** 2012-12

**Authors:** Rita Berisio, Luigi Vitagliano

**Affiliations:** Istituto di Biostrutture e Bioimmagini, CNR via Mezzocannone 16, I-80134 Napoli, Italy

**Keywords:** Collagen, triple helix, host-pathogen interaction.

## Abstract

Secondary structure elements often mediate protein-protein interactions. Despite their low abundance in folded proteins, polyproline II (PPII) and its variant, the triple helix, are frequently involved in protein-protein interactions, likely due to their peculiar propensity to be solvent-exposed. We here review the role of PPII and triple helix in mediating host-pathogen interactions, with a particular emphasis to the structural aspects of these processes. After a brief description of the basic structural features of these elements, examples of host-pathogen interactions involving these motifs are illustrated. Literature data suggest that the role played by PPII motif in these processes is twofold. Indeed, PPII regions may directly mediate interactions between proteins of the host and the pathogen. Alternatively, PPII may act as structural spacers needed for the correct positioning of the elements needed for adhesion and infectivity.

Recent investigations have highlighted that collagen triple helix is also a common target for bacterial adhesins. Although structural data on complexes between adhesins and collagen models are rather limited, experimental and theoretical studies have unveiled some interesting clues of the recognition process. Interestingly, very recent data show that not only is the triple helix used by pathogens as a target in the host-pathogen interaction but it may also act as a bait in these processes since bacterial proteins containing triple helix regions have been shown to interact with host proteins.

As both PPII and triple helix expose several main chain non-satisfied hydrogen bond acceptors and donors, both elements are highly solvated. The preservation of the solvation state of both PPII and triple helix upon protein-protein interaction is an emerging aspect that will be here thoroughly discussed.

## SECONDARY STRUCTURE AND PROTEIN-PROTEIN INTERACTIONS

Protein structure is usually described as a hierarchal assembly of constitutive building blocks [1]. The vast majority of folded proteins contain one or more stretches of amino acids that assume repetitive and characteristic structures in the three-dimensional space denoted as secondary structure elements. Statistical surveys of protein structure models have unveiled the existence of several secondary structure elements. The most common ones, each assumed by approximately the 30% of the residues in protein structures, are α-helix and β-structure [2-5]. Less common motifs are 3(10)- and π-helix, polyproline II (PPII) helices and a variant of PPII denoted as triple helix [6-15]. 

Secondary structure elements play a major role in the stabilization of protein folds. However, they may also be crucially involved in molecular recognition processes, such as protein-protein, protein-DNA, protein-RNA, protein-sugars interactions. The involvement of secondary structure elements in molecular recognition obviously depends on their location within the protein context. Helices, that are often solvent exposed, commonly mediate protein-protein contacts. The role of the β-sheets in these processes is more intricate being these structure elements highly reactive and typically located in the core of protein structure [1]. Nonetheless, they frequently mediate protein-protein interactions, although their reactivity, if uncontrolled, may lead to unwanted aggregation processes underlying several pathological states [16-21]. The role of α-helices and β-structures in molecular recognition has been comprehensively and effectively reviewed in previous reports [21,22]. Here, we focus our attention to the role that recognition processes mediated by PPII and triple helix with a specific emphasis in host-pathogen interactions. A particular attention will be given to the structural aspects related to these processes.

## POLYPROLINE HELICES: BASIC FEATURES

Proline is unique among genetically-encoded aminoacids [23]. The specific covalent link between the C^δ^ atom of the Pro side chain with the backbone nitrogen atom confers this residues peculiar conformational properties. As a result of its cyclic nature, Pro φ angle is restricted to values of -65°±15° [24-26]. In addition, the particular location of the C^δ ^atom also restricts the ψ value of the preceding residue [24-27]. As a consequence of this conformational restrictions, in proteins proline-rich regions are known to preferentially adopt an extended conformation denoted as called the poly-L-proline helix of type II (PPII). PPII helices are left-handed, all-trans extended helices characterized by (φ, ψ) backbone dihedral angles with average values of (-75°, 145°) [6-10,28]. These extended helices have three residues per turn, with a pitch value of 9.3 Å/turn. 

One of the distinctive structural properties of PPII helices, compared to the other common secondary structure elements, is the absence of local hydrogen bonding patterns involving main chain atoms. This feature leaves several non-satisfied hydrogen bond donors and acceptors free to establish intra- or inter-molecular interactions (see below). Furthermore, the absence of specific hydrogen bonding patterns makes the formation of long PPII helices rather unusual in globular proteins [6-10]. It has been proposed that the backbone conformations of unfolded proteins are not random coils but include short stretches of PPII structural motifs interspersed with turns and bends (polyproline hypothesis for unfolded proteins) [29-32]. The absence of regular hydrogen bonds makes of PPII helices dynamic structural entities whose plasticity may be important in protein-protein interaction [33].

One open question is the nature of the determinants of PPII stability, since it does not rely on hydrogen bonding interactions. In addition to the established entropic effects favoring the formation of PPII structures by iminoacids, one appealing novel proposal is that that an electronic effect of the n→p* interaction between the nonbonding n-orbital of the its peptide carbonyl oxygen atom and the anti-bonding p*-orbital of the (i-1)th peptide carbonyl carbon atom provides substantial stabilization to the left-handed PPII [34]. Given the typical exposure to the solvent of these helices [35], it is also possible that solvation plays an important role in PPII stabilization.

## POLYPROLINE HELICES IN HOST-PATHOGEN RECOGNITION

The peculiar propensity of both the side chain and the backbone atoms of the PPII helices to be solvent-exposed makes it an excellent recognition motif [35,36]. Indeed, proline-rich regions are predominantly localized in the solvent-exposed regions such as the loops, intrinsically disordered regions, or between domains that constitute the intermolecular interface. It is, therefore, not surprising that PPII helices are frequently involved in intermolecular interactions as signal transduction, antigen recognition, cell-cell communication and cytoskeletal organization. Peptide mimics of the PPII recognition motifs have been suggested as potential antagonists of intermolecular interactions [37].

The analysis of the energetic determinants of PPII binding to their structural cognates suggests that, in general, two main forces operate in concert. The first is the stacking of proline side-chains with the hydrophobic grooves in the binding site of PPII receptors whereas the second is represented by a firm network of water-mediated hydrogen bonding interactions. In this context, it is important to note that recent investigations have highlighted the role of hydration in PPII recognition. Current data indicate that the tendency of proline-rich sequences to be highly hydrated has been exploited by nature to favor the adaptability and plasticity of the different families of protein modules for the recognition of proline-rich targets [35]. It has been suggested that interfacial water molecules may provide important clues for deciphering the specificity versus promiscuity paradox in polyproline recognition [35].

As for many other biological processes, PPII motifs also play a role in host-pathogen recognition. One of the best characterized examples in this field is represented by the ability of PPII motifs of the protein ActA of the food-borne pathogen *Listeria monocytogenes* to bind the EVH1 domain of the mammalian protein Mena, an important factor in cytoskeleton regulation. 

The interaction of ActA with Mena EVH1 is the molecular event that allows the bacterium to utilize the actin cytoskeleton to move and infect neighboring cells [38-40]. Since the inhibition of this interaction may provide a strategy to hamper the motility of the pathogen and to prevent its spread in the host organism, many efforts are currently devoted to the development of peptides able to interfere with ActA/Mena interaction [41]. In this scenario many efforts are currently made to fully elucidate the structural and energetic details of this protein-protein interaction.

A direct involvement of PPII motifs in host-pathogen recognition has also been unveiled for virus infections. A prototypal example in this context is represented by the HIV-1 Nef, a multifunctional protein required for full pathogenicity of the virus [42]. Although the role of this pathogenic factor in the progression to AIDS is not mechanistically understood, it is commonly assumed that Nef necessarily functions through protein-protein interaction, as it does not present any enzymatic activity. It has been shown that the critical Nef protein interaction interface is centered on its polyproline segment (P69VRPQVPLRP78) which contains the helical SH3 domain binding protein motif, PXXPXR [43]. 

A PPII motif is also responsible for the activation of the phosphatidylinositol 3-kinase (PI3K)/Akt signaling pathway by the influenza A virus [44]. The activation of this pathway is beneficial for virus replication by inhibiting virus induced apoptosis through phosphorylation of caspase-9. Mutational studies have demonstrated that the influenza NS1 protein is able to bind the p85 regulatory subunit of PI3K though a PXXP region (amino acids 164 to 167) [45]. 

**Fig. (1):**
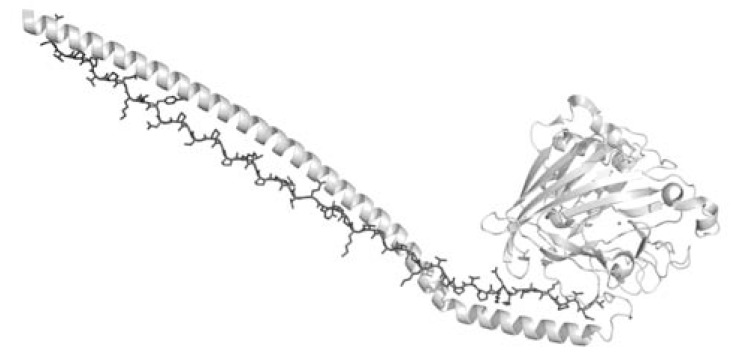
Representation of the interaction between the α-helix (light grey) and the PPII helix (dark grey) found in AgI/II structure (PDB code 3iox). For clarity, the PPII helix was reported as a stick model.

In addition to the direct involvement in protein-protein interactions occurring in infectious diseases, recent investigations suggest that PPII may facilitate host-pathogen interactions through other mechanisms. Indeed, PPII motifs may act as structural spacers needed for the correct positioning of the elements needed for adhesion and infectivity. One of the most striking examples of this possibility has been highlighted by the determination of the three-dimensional structure of the A3VP1 fragment of the adhesin antigen I/II (AgI/II) localized on the cell surface of the pathogen *Streptococcus mutans*, which is the causative agent of human dental caries. The crystallographic structure of this protein has unveiled a novel and unique structural motif, a hybrid structure composed of α- and PPII helices [46]. In particular, the A3 repeat of the alanine-rich domains of the protein adopts an extended α-helix that intertwines with the P1 repeat in PPII conformation, to form a fibrillar structure which is 155 Å long (Fig. **1**). Interestingly, this motif is stabilized by interactions that are commonly associated to PPII protein-protein recognition. In particular, these include: (a) hydrogen bonding of non-saturated main chain atom of PPII, (b) hydrophobic interaction of exposed proline side chains with aromatic residues, and (c) water mediated hydrogen bonding networks. It is important to emphasize that in the case of AgI/II, the PPII helix acts as a structural element that is crucial to the building of the functional architecture of an essential protein in the pathogen adhesion to the tooth surface [46,47].

Another recent example of a possible structural role for PPII motifs has emerged from the crystallographic characterization of the Group A streptococci basal pilin FctB, which presents an immunoglobulin-like N-terminal domain with an extended PPII tail [48]. This tail is a proline-rich fragment (residues 123–137) with sequence PXPPXXPXXPXXPXXP. Although terminal PPII fragments are expected to be high mobile, the PPII helix of FctB is structurally well-defined, as indicated by its surprisingly clear electron density. Although the precise role for this motif is yet to be clearly defined, the PPII helix likely acts as a “spacer” ensuring that a cell wall-anchored protein actually protrudes from the thick bacterial peptidoglycan (PGN), an essential bacterial cell wall polymer, formed by glycan chains of β-(1-4)-linked-N-acetylglucosamine and N-acetylmuramic acid cross-linked by short peptide stems. Attachment to the PGN is mediated by a region called LPXTG motif, which is cleaved between the Thr and Gly residues by a transpeptidase called sortase and the carboxyl group of Thr is covalently joined to the carboxyl group of a PGN peptide stem. In this context, the PPII helix seems to be important to separate the immunoglobulin-like domain from the LPXTG sortase cleavage motif and leave space for the sortase protein to act. 

The presence of a proline-rich C-terminal tail is not unique to pilin proteins and can be found in other cell wall-anchored proteins [49]. Because only one pilin protein per pilus operon appears to have a proline-rich tail, Baker and colleagues [48] have proposed that such domains preceding a sortase motif is a common structural element involved in cell wall anchoring.

## TRIPLE HELIX: BASIC STRUCTURAL FEATURES

Triple helix is a structural motif characterized by the association of three distinct polypeptide chains wrapped around a common axis [12-15]. Each of these chains assumes a PPII-like conformation. The structural features of the triple helices requires the presence of Gly residuesin the inner region of the helix, every three residues of the polypeptide sequences. Therefore, sequences adopting these structural elements are characterized by the repetition of triplets of the type Gly-Xaa-Yaa [12-15]. Although all types of amino acids may be located at positions X and Y of the triplets, they are frequently occupied by iminoacids [Pro and its post-translationally modified form 4R-hydroxy-2S-proline (4RHyp)]. The role of iminoacids in triple helix stabilization is a subject of intense research activities that has been reviewed and discussed in several literature reports [12-15,50-60]. 

Collagens are prototypal triple helix containing proteins. However, the triple-helical motif is also found in a variety of non-collagenous proteins, such as macrophage scavenger receptors types I and II and bacteria-binding receptor MARCO, the complement component C1q, pulmonary surfactant apoproteins A and D, acetylcholinesterase, bovine conglutinin, collectin-43, ficolins, aggretin, ectodysplasm, mannose binding protein and in several proteins isolated by invertebrates and bacteria (see below) [61].

The triple helix is stabilized by the formation of an intermolecular hydrogen bond between the nitrogen of the Gly residue and the carbonyl group of the residue located in the X position. Further contribution to the triple helix stabilization is occasionally provided by intermolecular interactions established by specifics side chains [12-15,62-66]. 

The non-globular shape of triple helix regions has hitherto prevented high resolution structural characterization of entire proteins carrying this structural motif [see for example ref [67,68]]. Available structural information on the triple helix have been essentially achieved through the characterization of peptide models in triple helix conformation [12-15,62,69]. High resolution studies carried out on these models have provided a detailed three dimensional picture of triple helix. Indeed, these investigations have unveiled a number of important structural details which have proven to be essential for a full understanding of triple helix stability and its possible interactions modes with solvent or other biological partners. As anticipated by initial triple helix models, in PPII side chains of residues located in positions X and Y of repetitive triplets are fully exposed to the solvent. Since these positions are often occupied by iminoacids (Pro or 4RHyp), whose side chain is partially aliphatic, one of the distinctive features of triple helix is the exposure of hydrophobic region that presents a strong propensity to participate to intermolecular interactions. High resolution studies have also shown (a) that the position X and Y of the triple helix are not conformationally equivalent and (b) triple helix geometrical parameters may locally change to generate helices with variable symmetries ranging from 7_5_ or to 10_7_. The implications of these latter findings have been extensively described in previous literature papers [12-15,62,69-75]. Crystallographic studies have also provided evidence that triple helix is highly hydrated. The first hydration shell is composed by waters that interact either with non-saturated main chain hydrogen bonding donors and acceptors (nitrogens and carbonyl oxygens) or with side chain hydrophilic groups. Usually, other layers of water molecules are bound to those of the first shell to generate complex networks that are able to connect different triple helices in the crystalline state.

In conclusion, the triple helix preserves some of the features of isolated PPII helices (solvent exposure of non-satisfied hydrogen bond acceptors or donors and of hydrophobic residues). The tight association of the three chains confers to this motif a structural rigidity that is missing in PPII helices. These features play a crucial role in triple helix interaction with other biological actors.

## MOLECULAR RECOGNITION OF COLLAGEN TRIPLE HELIX

Collagens are the most abundant proteins in mammals. The collagen family comprises 28 members that contain at least one triple-helical domain [76]. Collagens are deposited in the extracellular matrix where most of them generate supramolecular assemblies. Traditionally, collagens have been seen as structural entities that contribute to mechanical properties, to the organization and to the shape of tissues. More recent studies have highlighted that collagens are also involved in a variety of biological processes such as survival signals, morphogenic processes, and diseases. 

Despite its limited sequence variability and the simple rod-like shape, collagens are promiscuous proteins that are able to interact with several receptor families to perform their regulatory function in cell proliferation, migration, and differentiation. 

**Fig. (2):**
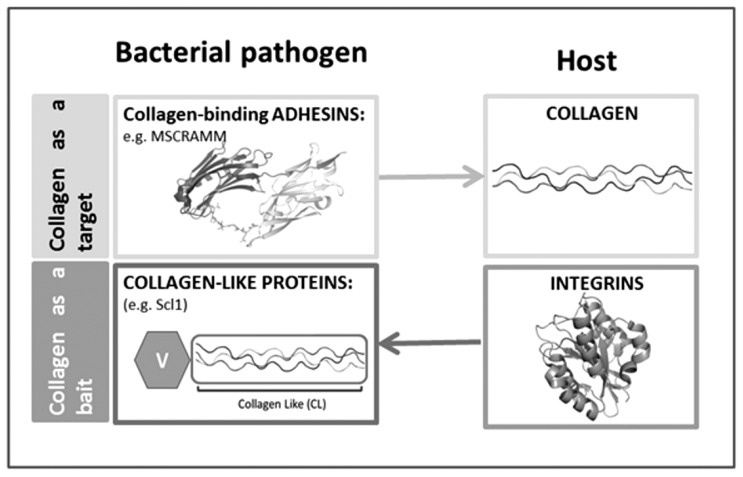
Scheme ‘collagen acts as a target and a bait’.

Collagen-interacting proteins are expressed by both eukaryotes and prokaryotes. Indeed, a number of transmembrane proteins, collectively designed as mammalian collagen–receptors [77], are able to interact with triple helical motifs. Interestingly, recent data show that not only is the triple helix used by pathogens as a target in the host-pathogen interaction but it may also act as a bait in these processes since bacterial proteins containing triple helix regions have been shown to interact with host proteins (Fig. **2**) (see below for details). 

Although a full understanding of the structural bases of the collagen recognition is yet to be achieved, collagen–receptor interaction generally occurs through two distinct mechanisms [77]. In some cases, as for example integrins and discoidin domain receptors and chaperone FKBP65, the receptor recognizes a specific collagen sequence. In other cases, as glycoprotein VI, CNA, YadA, saratin, and LAIR-1, the receptor simply recognizes and binds the basic triple helix motif [77-80]. 

**Fig. (3):**
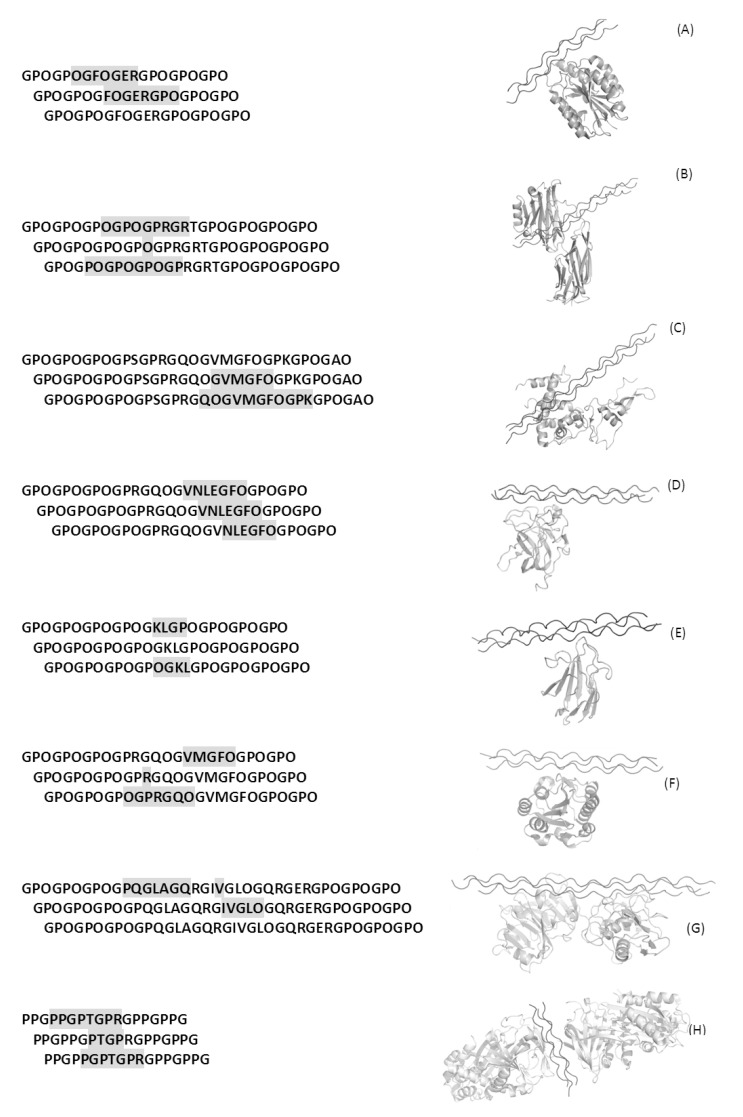
Crystal structures of complexes between proteins and collagen triple helices. Left panel reports sequences of bound triple helices: residues of the triple helices located within 4 Å from the receptor are reported in grey boxes. Right panels report ribbon representations of their three-dimensional structures. Panels A-G refer to complexes of triple helices with integrin alpha 2 I domain (PDB code 1dzi), CNA (2f6a), SPARC (2v53), DDR2 discoidin domain (2wuh), MASP-1 CUB2 domain (3pob), Von Willebrand factor A3 domain (4dmu), matrix metalloproteinase 1 (4auo), and the chaperone Hsp47/SERPINH1 (4awr) respectively.

Over the years a number of complexes involving triple helix peptides and their biological cognates have been elucidated by X-ray crystallography. So far the structure of seven complexes formed by triple helical peptides are reported in the Protein Data Bank (PDB). These complexes are formed with integrin alpha 2 I domain (PDB code 1dzi) [81], CNA (2f6a) [82], SPARC (2v53) [83], DDR2 discoidin domain (2wuh) [84], MASP-1 CUB2 domain (3pob) [85], Von Willebrand factor A3 domain (4dmu) [86], matrix metalloproteinase 1 (4auo) [87] and the chaperone Hsp47/SERPINH1 (4awr) [88]. These studies have provided important clues on collagen molecular recognition, although differing views have been reported for MMP1 binding [88,89]. With the exclusion of the collagen binding protein CNA isolated from *Staphylococcus aureus*, these triple helix interacting proteins are of mammalian origin. Notably, the mammalian proteins target a specific sequence region of the triple helix motif, whereas CNA targets a consensus motif (Gly-Pro-Hyp). In all cases, the interacting surface between these proteins and the triple helix is not particularly large and involves residues of at least two of the three chains of the triple helix (Fig. **3**). The specific features of the complex between triple helix and the protein CNA isolated from the pathogen *S. aureus* will be illustrated in the following sections.

## HOST COLLAGEN TRIPLE HELIX AS A TARGET FOR BACTERIAL PATHOGENS

Bacterial adherence to host tissues represents an early critical step in the infection process. Extracellular pathogens, such as enterococci, staphylococci, and streptococci, often target extracellular matrix components for attachment and colonization. Adherence is mediated by protein adhesins of the MSCRAMM (microbial surface components recognizing adhesive matrix molecules) family. These adhesive proteins from Gram-positive bacteria do not share sequence homology with the collagen binding I domains of integrins and do not require metal ions for collagen binding. Consistently, these proteins employ a collagen binding mechanism that is drastically different from that of the collagen binding integrins.

**Fig. (4):**
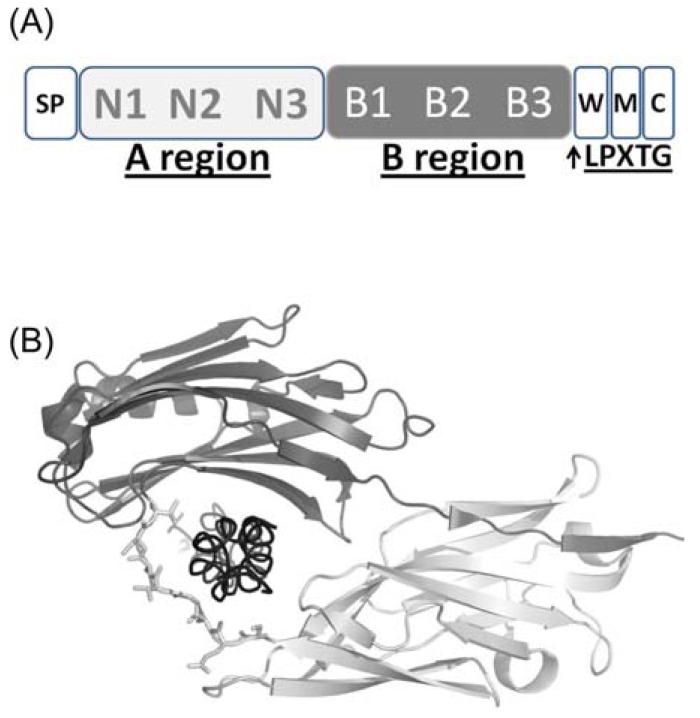
(A) domain organisation of MSCRAMM proteins CNA and ACE: The N-terminal signal peptide (SP) is followed by A and B regions, the C-terminal cell wall sorting region W, the transmembrane region M and the cytoplasmatic tail C. (B) CNA structure in complex with the collagen triple helix. The N1 and N2 domains of the A region are shown in light and dark gray, respectively. Collagen triple helix (black) is wrapped by the linker region between N1 and N2, represented in ball-and-stick.

These MSCRAMMs contain an N-terminal signal peptide followed by a non-repetitive region called the A region, which in most cases is responsible for ligand binding (Fig. **4**). The A regions are composed of two or more subdomains each adopting an immunoglobulin G-like (IgG-like) fold. Following the A region is often a segment composed of repeated sequences or motifs that is referred to as the B region [90]. In most cases, MSCRAMMs are covalently anchored to the cell wall peptidoglycan (PGN) by the sortase through its C-terminal LPXTG motif (Fig. **4**).

MSCRAMM proteins mediate bacterial attachment to the host through interactions with either host fibronectin or collagen triple helix. The collagen-binding protein CNA is responsible for adherence of *S. aureus* to collagen substrates and collagenous tissues. Consistently, antibodies against CNA inhibit bacterial binding to collagen and block bacterial adherence to cartilage [91]. CNA participates in the infectious process of pathogenic *S. aureus* and is shown to be a virulence factor in many different animal models of infections [92,93]. This suggests that the ability to interact with collagen provides a general advantage to the bacteria in pathogenesis.

So far, the structure of a single MSCRAMM complex (CNA from *S. aureus*) with its collagen ligand has been determined experimentally through X-ray crystallography [94-96]. Structure of unligated MSCRAMM s have also been reported, see for example [90,96]. Altogether, these structures have provided fundamental clues on collagen recognition by bacterial proteins mediated by specific or non-specific collagen sequence motifs. Also, they have highlighted differences compared to collagen binding integrins. Indeed, the crystal structure of the complex between the I domain of integrin α2β1 and a triple helical collagen peptide unveiled that a collagen glutamate completes the coordination sphere of the metal of the integrin [81]. A completely different mechanism, denoted as ‘collagen hug mechanism’ was proposed for collagen recognition by bacterial proteins. 

The crystal structure of CNA exhibits two distinct domains (called N1 and N2) (Fig. **4**). The N-terminal N1 domain exhibits an IgG-like fold, which contains two additional strands D’ and D’’ compared to the conventional IgG fold [94,97]. The N2 domain is similar to the previously determined crystal structure of CNA151–318 [98], which contains in addition to the D2’ and D2’’ strands, an extra D2’’’ strand and a two-turn α-helix. The N1 domain that is completed by the C-terminal extension of the N2 domain (Fig. **4B**). This organization creates a two-domain structure with a distinct hole between the two domains (Fig. **4B**). The collagen triple helix is seen penetrating through the hole, of about 12 to 15Å in diameter, between the N1 and N2 domains of can (Fig. **4B**). 

**Fig. (5):**
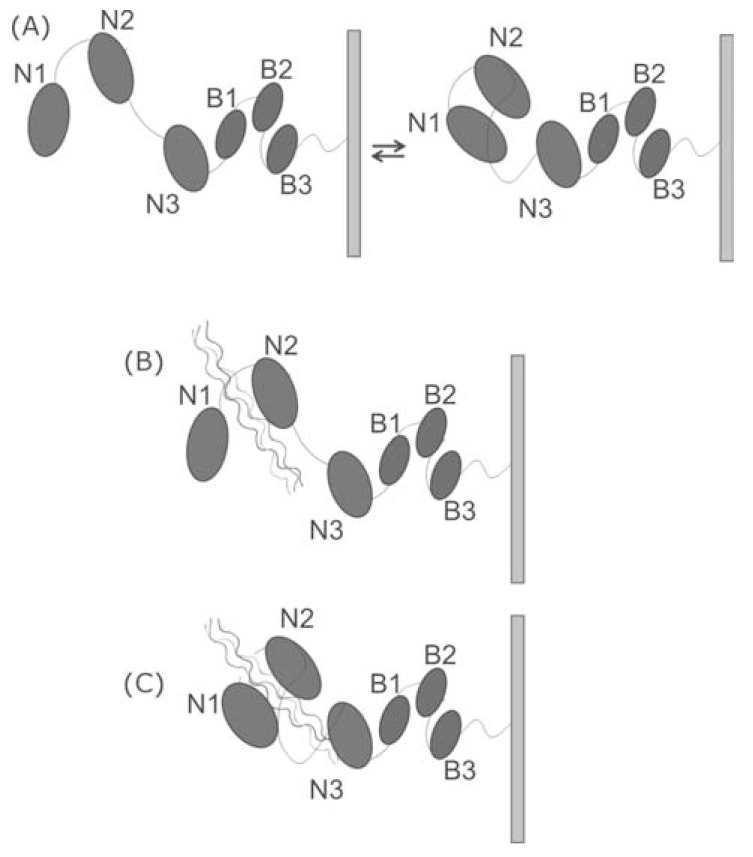
Collagen hug mechanism. (A), CNA open and close forms exist in equilibrium; (B) Collagen binds to the N2 domain; (C) collagen is wrapped by N1 and N2 domains and by the inter-domain linker.

Since the closed conformation cannot bind the collagen triple helix, the ‘Collagen Hug model’ proposes that the initial event of the collagen recognition requires that the apo-form exists in equilibrium between an open and closed conformation, and that only the open conformation can bind collagen (Fig. **5**) [95]. The following steps of this mechanism closely resemble those of the interaction mode reported for MSCRAMM recognition of fibrinogen, and denoted as the ‘Dock, Lock and Latch’ model (Fig. **5**). 

The binding is guided by sequential interactions of the collagen triple helix with the N2 domain with the interdomain linker and eventually with the repositioned N1 domain. The collagen peptide ligand is further secured by conformational changes of the linker region, which keeps the collagen triple helix tightly in place. Namely, MSCRAMM appears to wrap around the collagen triple helix where the N1 and N2 subdomains of CNA create a “tunnel-like” structure that “locks” the collagen ligand in between the two subdomains. In a final step, the C-terminal extension of the N2 subdomain acts as a “latch” by inserting into a trench present on the N1 subdomain by β-strand complementation [99]. Molecular dynamics simulations carried out on CNA suggest that the incomplete Ig-like fold of CNA N2 generated by the displacement of the C-terminal extension is sufficiently stable to support the Collagen Hug mechanism and also evidence a key role of collagen hydration in CNA recognition [100]. In particular, hydration maps of the CNA-collagen complex reveal the presence of several structured water molecules that mediate intermolecular interactions at the interface between the two proteins. These hydration sites feature long residence times, significant binding free energies, and a geometrical distribution that closely resembles the hydration pattern of the isolated collagen triple helix. Therefore, CNA recognizes the collagen triple helix as a hydrated molecule [100]. 

Structural studies of the MSCRAMM protein ACE, the first to be identified on E. faecalis [101], showed a structural organization similar to that of the Staphylococcus aureus collagen adhesin CNA [90]. These studies corroborated the ‘Collagen Hug’ model, as they showed that point mutants which were stabilizing a closed conformation (by a disulfide bond) did not bind type I collagen. In addition, they identified key residues for collagen binding activity.

Evidence that bacterial matrix-binding proteins are virulence factors has come from studying defective mutants in adherence assays. Mutants lacking the collagen-binding protein have reduced virulence in a mouse model for septic arthritis, suggesting that bacterial colonization is ineffective. Furthermore, the isolated ligand-binding domain of the fibrinogen, fibronectin and collagen receptors strongly blocks attachment of bacterial cells to the corresponding host proteins [102,103]. The key role of collagen-binding adhesins in bacterial infections has encouraged the development of strategies to specifically block the interaction of bacteria with matrix collagen by antagonist ligands. For example, recombinant adhesion fragments and polyclonal antibodies may inhibit bacterial adhesion to host cells to varying degrees [104]. Also, specific antibodies against high-affinity binding subsegments of the MSCRAMM protein ACM were the most effective at inhibiting *E. faecium* adherence to host collagen [105]. Further investigations are still needed to advance our understanding of the mechanisms of bacterial adhesion and ways to hamper them.

## BACTERIAL COLLAGEN TRIPLE HELIX AS A BAIT FOR HOST-PATHOGEN INTERACTION

Although type I is the most widespread collagen, invertebrates were shown to contain several other types of collagen genes [106]. More recently, collagen-like sequences have been identified also in prokaryotic genomes [107-111]. These collagen-like proteins have been shown to adopt a triple helix structure, with a thermal stability similar to that seen for human collagens [112-114]. However, unlike mammalian collagens, prokaryotic collagens do not contain hydroxyproline, since bacteria lack the prolyl-hydroxylase enzyme necessary for post-translational modification of Pro to Hyp. 

**Fig. (6):**
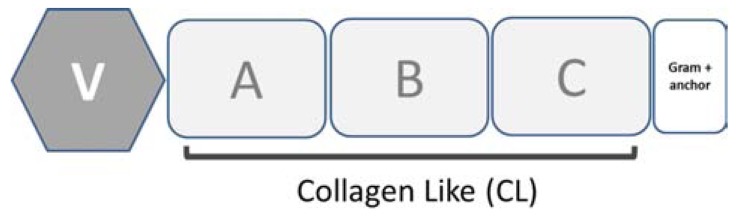
Domain organization of Scl proteins main features are: an N-terminal globular (V) domain, a collagen-like (CL) domain, and a C-terminal Gram-positive cell wall attachment domain (Gram+ anchor).

Best characterized prokaryotic collagens are the two collagen-like proteins, Scl1 and Scl2, which have been demonstrated to be simultaneously expressed on the cell surface of *Streptococcus pyogenes* and to be able to promote bacterial adhesion to the host [107,108]. Both Scl1 and Scl2 proteins contain a signal sequence, an N-terminal variable globular domain (V), a highly charged collagen-like triple-helix domain (CL) consisting of (Gly-Xaa-Yaa)n triplet repeats and a C-terminal Gram-positive cell wall attachment domain (Fig. **6**). The Scl1 and Scl2 proteins form stable triple-helical structures when expressed as recombinant proteins [115,116], and their N-terminal globular V domain adjacent to the triple-helix domain appears to be important for efficient triple-helix assembly [116, 117]. In *Streptococcus pyogenes* the CL domain can be dissected into three fragments of almost equal size with distinctive amino acid features (A, B and C). Fragment A has the highest content of polar residues, fragment B the highest Pro content, and fragment C the highest charged residue content. 

Bacterial collagen-like proteins [114] contain no Hyp, and their Pro residues are found preferentially in the Xaa positions, with the frequency of Pro residues in the X position exceeding 30%, whereas the frequency of Pro in the Y position is 5%. Despite the lack of Hyp, however, bacterial collagen-like proteins form triple-helix structures with a stability near 37 °C, close to that seen for mammalian collagens. In theory, bacteria could use Gly-Pro-Pro tripeptide units, which are highly stabilizing sequences, but Gly-Pro-Pro tripeptides are rarely found in bacterial collagen-like proteins. Interestingly, almost all bacterial collagens studied so far have melting temperatures between 36 and 38 °C, indicating that this narrow range of melting temperatures might have been evolutionarily set for host-pathogen interaction [112-114]. To explore the basis of bacterial collagen triple-helix stability in the absence of Hyp, biophysical studies were carried out on recombinant Scl2 protein and a set of peptides modeling the Scl2 highly charged repetitive (Gly-X-Y)n sequences [116]. These studies showed that triple helix stabilization is highly pH dependent, and that the Scl2 protein uses a variety of electrostatic interactions, inter-chain hydrogen bonds, and a hydration-mediated hydrogen bonding network as an alternative to the Hyp stabilization in animal collagens [116].

Scl proteins are key to host-pathogen recognition. It has been demonstrated that Scl1 can bind selected human extracellular matrix components [109], cellular integrin receptors [107,118,119], and plasma components [120-122]. Importantly, human collagen receptors such as integrins α2β1 and α11β1 recognize the triple helix CL domain of Scl1, and this event results in cell signaling [107,118,119]. This finding is a strong indication that collagen-like bacterial proteins display not only structural but also functional similarities to human collagens [107-110]. 

## FINAL CONSIDERATIONS

A survey literature data clearly indicates that PPII and triple helix motifs are able to establish interactions with a plethora of biological partners, despite their rather low sequence variability. Indeed, the over-representation of proline and proline derivatives in their sequences does not preclude to these elements the possibility to regulate diversified biological processes including those related to infections by external pathogens. It is important to note both PPII and triple helices exploit common features to establish protein-protein interactions. The first one is represented by the presence in both elements of exposed and non-satisfied main chain hydrogen bonding donor and acceptor groups. These groups may either directly bind the interacting proteins or firmly bind water molecules that, in turn, make hydrogen bonding interactions with the partner. One of the emerging ideas in the fields is that the hydration pattern of the isolated partners is, at least partially, preserved upon the formation of complexes involving PPII and triple helix. In near future, computational approaches, as those currently used for small molecules [123], may likely provide (a) interesting structural insights into these processes and (b) quantitative estimates of the energetics of water binding. The second feature of these elements is the exposure of hydrophobic side chains (especially proline residues) that are easily recognized by hydrophobic patches present on the receptor. The combination of these hydrophilic and hydrophobic patches in PPII and triple helix likely regulates the specificity of their partner recognition. It may also explain the paradox between specificity versus promiscuity of triple helix and PPII recognition. 

In this scenario, the specific assembly of PPII helices in the more structurally rigid triple helix preserves the exposure of the main reactive groups of this motif. The combination of rigidity and solvent-exposure of reactive groups makes triple helix suitable for both structural and recognition roles. Finally, it should be noted that despite the emerging interest in host-pathogen recognition processes mediated by either PPII or the triple helix, the amount of structural information available is still limited. More detailed information about receptor-binding capabilities and mechanisms of different collagen-like bacterial and mammalian proteins will help us to exploit these interactions for therapeutic purposes.

## ABBREVIATIONS

PPII: = Polyproline II
PDB: = Protein Data Bank
